# A molecular survey of vector-borne pathogens and haemoplasmas in owned cats across Italy

**DOI:** 10.1186/s13071-020-3990-x

**Published:** 2020-04-21

**Authors:** Maria Stefania Latrofa, Roberta Iatta, Federica Toniolo, Tommaso Furlanello, Silvia Ravagnan, Gioia Capelli, Bettina Schunack, Bruno Chomel, Andrea Zatelli, Jairo Mendoza-Roldan, Filipe Dantas-Torres, Domenico Otranto

**Affiliations:** 1 Department of Veterinary Medicine, University of Bari, Valenzano, Bari, Italy; 2 Istituto Zooprofilattico Sperimentale delle Venezie, Legnaro, Padova, Italy; 3 San Marco Veterinary Clinic and Laboratory, Veggiano, Padova, Italy; 4 Bayer Animal Health GmbH, Leverkusen, Germany; 5 Department of Population Health and Reproduction, School of Veterinary Medicine, University of California, Davis, USA; 6 Department of Immunology, Aggeu Magalhães Institute, Fundação Oswaldo Cruz (Fiocruz), Recife, Brazil; 7 Department of Pathobiology, Faculty of Veterinary Science, Bu-Ali Sina University, Felestin Sq., Hamedan, Iran

**Keywords:** Cat, Vector-borne pathogens, Zoonosis, Haemoplasmas, *Bartonella* spp., *Leishmania infantum*, Feline leukemia virus, Feline immunodeficiency virus

## Abstract

**Background:**

Feline vector-borne pathogens (FeVBPs) have been increasingly investigated for their impact on cat health and their zoonotic potential. The aim of the present study was to assess the prevalence of FeVBPs and haemoplasmas in cats across Italy and to identify potential risk factors linked to their occurrence.

**Methods:**

Blood samples from 958 owned cats living in the North (*n* = 556), Centre (*n* = 173) and South (*n* = 229) of Italy were tested for *Babesia* spp., *Hepatozoon* spp., *Ehrlichia* spp., *Anaplasma* spp. and filarioids by conventional PCR (cPCR) and for haemoplasmas and *Bartonella* spp. by SYBR green real-time PCR. Cats included in the study represent a sub-sample from a larger number of animals enrolled in a previous study, which were selected based on the geographical origin. Data on cats’ positivity for *Leishmania infantum*, feline leukaemia virus (FeLV) and for feline immunodeficiency virus (FIV), available from the previous study, were included and examined. Potential risk factors for pathogen infection were assessed in relationship to categorical variables including sex, geographical origin, breed, neutering status and age of cats.

**Results:**

Out of the 958 cats, 194 (20.2%) were positive for at least one of the tested pathogens, 89 (16%) from the North, 32 (18.5%) from the Centre and 73 (31.9%) from the South of Italy. A high prevalence of FeVBPs was detected in male cats (*n* = 125, 27.8%), living in the southern part of the country (*n* = 73, 31.9%), younger than 18 months of age (*n* = 24, 22.4%) and not neutered (n = 39; 27.5%). In particular, 24 cats (2.5%) tested PCR-positive for *Bartonella* spp., of which 1.6% for *B. henselae* and 0.9% for *B. clarridgeiae*. A total of 111 cats scored PCR-positive for haemoplasmas (11.6%), specifically “*Candidatus* Mycoplasma haemominutum” (*n* = 95, 9.9%), *M. haemofelis* (*n* = 14, 1.5%) and “*Candidatus* Mycoplasma turicensis” (*n* = 2, 0.2%). Moreover, 39, 31 and 8 cats were positive for FeLV (4.1%), *L. infantum* (3.2%) and FIV (0.8%), respectively. Co-infections were registered for 19 (9.8%) cats.

**Conclusions:**

These results confirm the occurrence of haemoplasmas and FeVBPs throughout Italy. Preventive measures to protect both animal and human health should be carried out also for owned cats, even if no health status of animals has been assessed in this study.
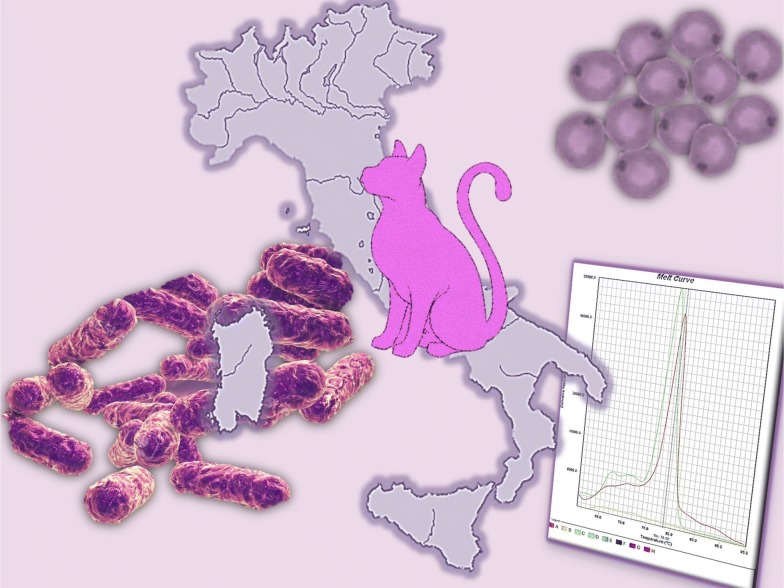

## Background

Vector-borne diseases (VBDs), caused by pathogens and transmitted by invertebrate vectors to vertebrate hosts, may represent a relevant health issue for pet animals and humans, considering the close association among them [1]. However, whilst VBDs are widely recognized in dog populations worldwide, cats are considered to be less frequently affected [2–4]. The sub-clinical and non-specific clinical signs and laboratory abnormalities of feline VBDs (FeVBDs) [2, 5] may further contribute to the underestimation of their relevance, reflecting in a paucity of data about these diseases [4, 6, 7]. In addition, even if some cat habits, such as grooming, may minimize the success of ectoparasite infestation, their outdoor lifestyle increases the exposure to arthropod vectors, and consequently, to their transmitted pathogens [2, 8–11].

Most of the literature generated on FeVBDs worldwide has been focussed on feline leishmaniosis in concomitant with viral infections by feline leukaemia virus (FeLV) and feline immunodeficiency virus (FIV) and on feline bartonellosis or haemoplasmosis [3, 12–17]. In contrast, a relative low number of studies were conducted on *Rickettsia* spp., *Anaplasma* spp., *Hepatozoon* spp. and *Babesia* spp. [18–21]. FeVBDs have been reported in cat populations in different countries of the Mediterranean basin (e.g. Cyprus, Greece, Spain and Italy) and in Portugal, with large variability in their prevalence due to different diagnostic techniques employed (i.e. serological and/or molecular tests), the animals’ lifestyle (i.e. indoor, outdoor) as well as the sample size tested [5, 11, 15, 18, 20–27]. These methodological differences make it difficult to draw comparisons for FeVBDs prevalence and to achieve a complete picture for areas such as the Italian Peninsula. Therefore, the aim of this study was to obtain data on the prevalence of feline vector-borne pathogens (FeVBPs) and haemoplasma infections in privately owned cats from different Italian regions using a comprehensive molecular methodology, and to assess the potential role of cats as reservoirs and potential sources of microorganisms that could be transmitted to humans.

## Methods

### Animal enrolment

Feline blood samples (*n* = 958) were received from veterinary analysis laboratories after animal’s health check, from different regions of the North (*n* = 556, Friuli Venezia Giulia, Liguria, Lombardy, Piedmont, Trentino Alto Adige, Valle D’Aosta, Veneto), Centre (*n* = 173, Emilia Romagna, Lazio, Tuscany, Marche, Umbria) and South (*n* = 229, Abruzzo, Calabria, Campania, Apulia, Sicily, Sardinia) of Italy (Table 1). Cats included in the study represent a sub-sample from a larger number of animals enrolled in a previous study [17] selected based on the geographical origin. For each region, all blood samples were chosen if cats were less than 15 or only 30% of them when over 15 animals. Within each region, cats were selected using computer-generated random numbers. All cats were examined according to age (less than 18 months-old, between 18 months and 6 years-old, and more than 6 years-old), sex, neutering status, breed and geographical origin (North: N; Centre: C; South: S) (Table 1). Data on cats’ positivity for *L. infantum*, FeLV and FIV, available from the previous study [17], were included and analysed (Table 1). For all cats included, no information on their health status and on ectoparasitic treatment were available.

**Table 1:** Prevalence of FeVBPs, haemoplasmas, FIV and FeLV infection accordingly to age, sex, breed and reproductive status of cats across Italy

Variable	*n*	Species												Total Pos (%)
		*Bartonella* spp.			Haemoplasmas				*Leishmania infantum*			FIV	FeLV	
		*B. h.* Pos (%)	*B. c.* Pos (%)	Total Pos (%)	CMhm Pos (%)	CMt Pos (%)	Mhf Pos (%)	Total Pos (%)	qPCR Pos (%)	IFAT Pos (%)	Total Pos (%)	Pos (%)	Pos (%)	
Age														
< 18 months	107	5 (4.7)^a, b^	4 (3.7)^c^	9 (8.4)^d, e^	7 (6.5)	–	2 (1.9)	9 (8.4)	–	1 (0.9)	1 (0.9)	–	6 (5.6)	24 (22.4)
18 months ≤ 6 years	242	2 (0.8)^a^	2 (0.8)	4 (1.6)^d^	22 (9.1)	–	4 (1.7)	26 (10.7)	1 (0.4)	9 (3.7)	9 (3.7)	2 (0.8)	14 (5.8)	50 (20.1)
> 6 years	609	8 (1.3)^b^	3 (0.5)^c^	11 (1.8)^e^	66 (10.8)	2 (0.3)	8 (1.3)	76 (12)	6 (1)	16 (2.9)	21 (3.4)	6 (0.9)	19 (3.2)	120 (19.7)
Sex														
Male	446	7 (1.6)	4 (0.9)	11 (2.5)	71 (16)^f^	–	7 (1.6)	78 (17.5)^g^	5 (1.1)	17 (3.8)	22 (4.9)^h^	8 (1.8)	22 (4.9)	125 (27.8)^i^
Female	512	8 (1.6)	5 (1)	13 (2.5)	24 (4.7)^f^	2 (0.4)	7 (1.4)	33 (6.4)^g^	2 (0.4)	9 (1.7)	9 (1.7)^h^	–	17 (3.3)	69 (13.5)^i^
Neutering status														
Neutered	816	12 (1.5)	6 (0.7)	18 (2.2)	81 (9.9)	2 (0.2)	11 (1.3)	94 (11.5)	7 (0.8)	17 (2.1)^j^	22 (2.7)^k^	4 (0.5)^l^	31 (3.8)	155 (19)^m^
Not neutered	142	3 (2.1)	3 (2.1)	6 (4.2)	14 (9.8)	–	3 (2.1)	17 (12)	–	9 (6.3)^j^	9 (6.3)^k^	4 (2.8)^l^	8 (5.6)	39 (27.5)^m^
Breed*														
Common European	842	14 (1.7)	8 (0.9)	22 (2.6)	90 (10.7)	2 (0.2)	14 (1.7)	106 (12.6)	6 (0.7)	24 (2.8)	28 (3.3)	8 (0.9)	38 (4.5)	184 (21.8)
Persian	28	–	–	–	–	–	–	–	1 (3.6)	1 (3.6)	2 (7.1)	–	–	2 (7.1)
Siamese	17	1 (5.9)	–	1 (5.9)	1 (5.9)	–	–	1 (5.9)	–	1 (5.9)	1 (5.9)	–	–	2 (11.8)
Maine Coon	16	–	–	–	2 (12.5)	–	–	2 (12.5)	–	–	–	–	–	2 (12.5)
Chartreux	9	–	1 (11.1)	1 (11.1)	–	–	–	–	–	–	–	–	–	1 (11.1)
Scottish Fold	2	–	–	–	–	–	–	–	–	–	–	–	1 (50)	1 (50)
Korat	1	–	–	–	1 (100)	–	–	1 (100)	–	–	–	–	–	1 (100)
Sphynx	1	–	–	–	1 (100)	–	–	1 (100)	–	–	–	–	–	1 (100)
Geographical origin														
North	556	5 (0.9)^n^	2 (0.4)^o^	7 (1.3)^p^	51 (9.2)	2 (0.4)	6 (1.1)	59 (10.6)	1 (0.2)^r, s^	8 (1.4)^t^	9 (1.6)^v^	4 (0.7)	23 (4.1)	89 (16)^x^
Centre	173	2 (1.2)	1 (0.6)	3 (1.7)^q^	20 (11.6)	–	2 (1.1)	22 (12.7)	3 (1.7)^r^	1 (0.6)^u^	3 (1.7)^w^	1 (0.6)	5 (2.9)	32 (18.5)^y^
South	229	8 (3.5)^n^	6 (2.6)^o^	14 (6.1)^p, q^	24 (10.5)	–	6 (2.6)	30 (13.1)	3 (1.3)^s^	17 (7.4)^t, u^	19 (8.3)^v, w^	3 (1.3)	11(4.8)	73 (31.9)^x, y^
Total animals	958	15 (1.6)	9 (0.9)	24 (2.5)	95 (9.9)	2 (0.2)	14 (1.5)	111 (11.6)	7 (0.7)	26 (2.7)	31 (3.2)	8 (0.8)	39 (4.1)	194 (20.2)

*Note*: Significant differences in pathogens’ prevalence are marked with equal superscript letters. *P*-value < 0.05. * Only breed of cats scored positive for pathogens are listed

*Abbreviations*: n, number of animals; pos., positive; *B. h.*, *Bartonella henselae*; *B. c*., *Bartonella clarridgeiae*; CMhm, “*Candidatus* Mycoplasma haemominutum”; CMt, “*Candidatus* Mycoplasma turicensis”; Mhf, *Mycoplasma haemofelis*

### Sample collection and molecular procedures

From each cat, 2 ml of whole blood were collected by cephalic or jugular venipuncture into vacuum tubes EDTA and preserved at -20 °C until molecular processing. DNA was extracted from blood using the GenUP Blood DNA Kit (Biotechrabbit, Berlin, Germany), following the manufacturerʼs recommendations. All DNA samples were tested for *Babesia* spp., *Hepatozoon* spp., *Ehrlichia*/*Anaplasma* spp., filaroids, haemoplasmas and *Bartonella* spp. (Table 2). Molecular detection of *Babesia* spp., *Hepatozoon* spp., *Ehrlichia*/*Anaplasma* spp. and filarioids was performed by conventional PCR (cPCR) using primers targeting partial *18S* rRNA gene, *16S* rRNA gene and cytochrome *c* oxidase subunit 1 (*cox*1) gene, respectively (Table 2) [28–30]. Haemoplasmas and *Bartonella* spp. detection was performed by the SYBR green real-time PCR using primers and run protocols previously described (Table 2) [31, 32].

**Table 2:** Primers and target genes used for pathogen detection in cats across Italy

Pathogens	Primer sequence (5′–3′)	Target gene	Amplicon size (bp)	References
Haemoplasmas	^a^Mycf: AGCAATRCCATGTGAACGATGAA	*16S* rRNA	127	[31]
	^a^Mycr1: TGGCACATAGTTTGCTGTCACTT			
Haemoplasmas	^b^MycE929f: ACGGGGACCTGAACAAGTGGTG	*16S* rRNA	259	[26]
	^b^MycE1182r: AGGCATAAGGGGCATGATGACTTG			
*Mycoplasma haemofelis/M. haemocanis*	^b^RNasePF1: CTGCGATGGTCGTAATGTTG	RNaseP	166	[33]
	^b^RNasePR1: GAGGAGTTTACCGCGTTTCA			
*Bartonella henselae/B. clarridgeiae*	BART-LC-GEN-F: ATGGGTTTTGGTCATCGAGT	Citrate synthase	190	[32]
	BART-LC-HEN-R: AAATCGACATTAGGGTAAAGTTTTT			
	BART-LC-CLA-R: CAAGAAGTGGATCATCTTGG			
*Ehrlichia* spp./*Anaplasma* spp.	EHR16SD: GGTACCYACAGAAGAAGTCC	*16S* rRNA	345	[29]
	EHR16SR: TAGCACTCATCGTTTACAGC			
*Babesia* spp./*Hepatozoon* spp.	RLBF: GAGGTAGTGACAAGAAATAACAATA	*18S* rRNA	460	[28]
	RLBR: biotin-TCTTCGATCCCCTAACTTTC			
Filarioids	NTF: TGATTGGTGGTTTTGGTAA	*cox*1	660	[30]
	NTR: ATAAGTACGAGTATCAATATC			

^a^Primers used in real-time PCR for haemoplasma detection and differentiation

^b^Primers used in conventional PCR for haemoplasma detection and differentiation

*Bartonella* amplification products were directly sequenced for species identification, whilst haemoplasma-positive samples were amplified by cPCR with primers to allow the sequencing [26] and with primers for the differentiation between *Mycoplasma haemofelis* and *Mycoplasma haemocanis* [33] (Table 2). Amplified PCR products were visualized by gel-electrophoresis in 2% agarose gels containing Gel Red nucleic acid gel stain (VWR International PBI, Milan, Italy) and were documented in Gel Logic 100 gel documentation system (Kodak, New York, USA). All PCR products were purified and sequenced in both directions using the same forward and reverse primers, employing the Big Dye Terminator v.3.1 chemistry in a 3130 Genetic analyzer (Applied Biosystems, California, USA) in an automated sequencer (ABI-PRISM 377). Nucleotide sequences were aligned and analysed using Geneious platform version 9.0 (Biomatters Ltd., Auckland, New Zealand) [34] and compared with available sequences in the GenBank database using Basic Local Alignment Search Tool (BLAST; http://blast.ncbi.nlm.nih.gov/Blast.cgi). For all PCR runs, DNA of pathogen-positive and negative blood samples served as controls.

### Statistical analysis

Possible associations between infections and variables were assessed through univariate analysis while the eventual risk factors for *Bartonella* spp. and haemoplasmas were assessed through multivariate analysis. Exact binomial test established confidence intervals (CI) with 95% confidence level. The Chi-square test was used to compare percentages of positivity among categories of the same independent variables as well as the total prevalence of each agent.

For multivariate analysis different logistic regression models were performed using as dependent variable *Bartonella* spp. or haemoplasma positivity at each time and as independent categorical variables the following: sex, geographical origin (North, Centre and South), breed (European *vs* others), reproductive status (neutered or not), positivity to other pathogens and as a numerical variable, the increasing age. Co-linearity among independent variables was preliminarily assessed using Pearsonʼs correlation coefficient. A *P*-value < 0.05 was considered as statistically significant. Statistical analysis was performed using StatLib and SPSS for Windows (version 13.0, SPSS, Inc., Chicago, IL, USA).

## Results

Out of the 958 cats, 194 (20.2%; 95% CI: 17.8–22.9%) were positive for at least one FeVBP. Of those, 89 (16%, 95% CI: 13.1–19.3%) came from the North, 32 (18.5%, 95% CI: 13.2–25.1%) from the Centre and 73 (31.9%, 95% CI: 26.1–38.2%) from the South of Italy. A statistically significant difference in pathogen prevalence of infection was detected for male cats (*n* = 125, 28%, 95% CI: 24.0–32.4%, *χ*^2^ = 31.2, *df* = 1, *P* < 0.0001), not neutered cats (*n* = 39, 27.5%, 95% CI: 20.7–35.5%, *χ*^2^ = 5.4, *df* = 1, *P* = 0.02) and cats living in southern Italy (*n* = 73, 31.9%, 95% CI: 26–38.2%, N *vs* S: *χ*^2^ = 24.9, *df* = 1, *P* < 0.0001; C *vs* S: *χ*^2^ = 9.1, *df* = 1, *P* = 0.002).

A high prevalence of infection was detected in cats younger than 18 months-old (*n* = 24, 22.4%, 95% CI: 15.3–31.3%) (Table 1). In particular, 24 cats (2.5%; 95% CI: 1.7–3.7%) tested positive for *Bartonella* spp. with *Bartonella henselae* being the most common species found (*n* = 15, 1.6%; 95% CI: 0.9–2.3%) followed by *Bartonella clarridgeiae* (*n* = 9, 0.9%, 95% CI: 0.5–1.8%).

Among *Bartonella* species, a significant difference in prevalence was recorded between age groups (< 18 months *vs* 18 months < 6 years: *χ*^2^ = 9.5, *df* = 1, *P* = 0.002 and *vs* ≥ 6 years: *χ*^2^ = 14.6, *df* = 1, *P* < 0.0001, respectively) and geographical areas of provenance (N *vs* S: *χ*^2^ = 9.5, *df* = 1, *P* < 0.0001; C *vs* S: *χ*^2^ = 9.5, *df* = 1, *P* = 0.03) (Table 1).

For *B. henselae*, a significantly higher prevalence was registered for cats below 18 months compared to those above 18 months of age (*vs* 18 months < 6 years of age: *χ*^2^ = 5.6, *df* = 1, *P* = 0.02 and *vs* ≥ 6 years: *χ*^2^ = 5.8, *df* = 1, *P* = 0.016), whilst for *B. clarridgeiae* a significant difference in prevalence was recorded between cats below 18 months compared to those above 6 years of age (*χ*^2^ = 1.0, *df* = 1, *P* = 0.002) (Table 1).

A total of 111 cats were positive for haemoplasmas (11.6%; 95% CI: 9.7–11.8%) with a significant difference in prevalence between males and females (*χ*^2^ = 26.9, *df* = 1, *P* = 0.05) (Table 1) but not between age groups and geographical areas of provenance. For haemoplasmas, the highest prevalence was recorded for “*Candidatus* Mycoplasma haemominutum” (*n* = 95, 9.9%; 95% CI: 8.1–12.0%), with a statistically significant difference recorded between sexes (*χ*^2^ = 33.7, *df* = 1, *P* < 0.0001), followed by *M. haemofelis* (*n* = 14, 1.5%, 95% CI: 0.86–2.4%) and “*Candidatus* Mycoplasma turicensis” (*n* = 2; 0.2%, 95% CI: 0.04–0.76%) (Table 1). A prevalence of infection of 4.1% (*n* = 39), 3.2% (*n* = 31) and of 0.2% (*n* = 8) were registered for FeLV, *L. infantum* and FIV, respectively (Table 1).

A statistically significant difference in prevalence was recorded for *L. infantum* infection between males and females cats (*χ*^2^ = 7.7, *df* = 1, *P* = 0.006), for the neutering status (*χ*^2^ = 5.1, *df* = 1, *P* = 0.02) and for cats living in southern Italy (N *vs* S: *χ*^2^ = 21, *df* = 1, *P* < 0.0001; C *vs* S: *χ*^2^ = 8.2, *df* = 1, *P* = 0.004). Similarly, a statistically significant difference was recorded for the neutering status of cats positive for FIV (*χ*^2^ = 7.9, *df* = 1, *P* = 0.005) (Table 1).

Co-infections were found in 19 (9.8%) cats, specifically co-infections with more than two pathogens were recorded in four cats positive for “*Ca.* Mycoplasma haemominutum” + *L. infantum* + FIV + FeLV (*n* = 1), *M. haemofelis+L. infantum* + FIV + FeLV (*n* = 1), “*Ca*. Mycoplasma haemominutum” + *B. henselae* + *L. infantum* (*n* = 1) and with *M. haemofelis* + *L. infantum* + FIV (*n* = 1). No DNA of *Ehrlichia/Anaplasma* spp., *Babesia* spp., *Hepatozoon* spp. and filarioids was amplified.

The risk factor analysis revealed that cats from southern Italy were more likely to be positive for *Bartonella* spp. (ExpB = 2.500) but not for haemoplasmas. Male sex, older age and FIV positivity were risk factors for haemoplasmas and not for *Bartonella* spp. (Table 3). With the exception of FIV, no other co-infection resulted as risk factor for *Bartonella* spp. and haemoplasmas, respectively.

**Table 3:** Significant risk factors (ExpB) for *Bartonella* spp. and haemoplasmas in cats across Italy

Independent variable	B	SE	Wald	*df*	*P*-value	Exp (B)	95% CI for Exp (B)
Cats from South	*Bartonella* spp. risk factors						
	0.916	0.336	7.444	1	0.006	2.500	1.294–4.828
	Haemoplasma risk factors						
Sex (male *vs* female)	0.560	0.113	24.388	1	0.000	1.751	1.402–2.187
Increasing age	0.339	0.110	9.509	1	0.002	1.404	1.132–1.742
FIV positive	2.177	0.913	5.681	1	0.017	8.823	1.472–52.861

*Note*: Variables entered on the models at step 1: provenance (NCS), reproductive status, breed, sex, age, FIV and FeLV positivity. *Bartonella* and haemoplasmas positivity were entered as independent variable in each model

*Abbreviations*: B, estimated coefficient; SE, standard error; Wald, Wald statistic; df, degrees of freedom; P, significance value; Exp (B), predicted change in odds for a unit increase in the predictor

Blast analysis of representative sequences showed a nucleotide identity of 99–100% with those of *Bartonella* spp. and haemoplasmas available on GenBank (*B. clarridgeiae*: GU056189; *B. henselae*: KX499328; “*Ca.* Mycoplasma haemominutum”: EU839980; “*Ca.* Mycoplasma turicensis”: KR905457 and *M. haemofelis*: EU078617).

## Discussion

The molecular detection of FeVBPs and haemoplasmas carried out in this study allowed to estimate the presence of pathogens within a large population of cats across Italy, with “*Ca*. Mycoplasma haemominutum” (9.9%) being the most prevalent pathogen in cats, followed by *L. infantum* (3.2%), *B. henselae* (1.6%), “*Ca*. Mycoplasma haemofelis” (1.5%) and *B. clarridgeiae* (0.9%). For the other FeVBPs investigated, the overall prevalence of infection for *Bartonella* spp. (2.5%) is similar to that detected in a previous molecular investigation in cats with an indoor lifestyle from the South and Centre of Italy (4.8%) [11].

However, these data are in contrast with the prevalence for *Bartonella* spp. molecularly (up to 38.1%) [18] and serologically (up to 48.7%) detected in outdoor or free-roaming cats with ectoparasite infestation [4, 11, 15, 18, 21]. A similar high seroprevalence was reported in Spain (50%) [35] and Greece (58.8%) [23]. The higher prevalence for *B. henselae* (1.6%) compared to *B. clarridgeiae* (0.9%) is not surprising, as domestic cats are considered to be the main reservoir for *B. henselae*, the causative agent of cat-scratch disease [13, 36]. The same difference of infection rates between *B. henselae* and *B. clarridgeiae* has been reported in cat populations from southern and insular regions of Italy [18], even if, a higher level of infection rate was registered for both *Bartonella* species (up to 21.4% for *B. henselae* and 16.6% for *B. clarridgeiae*), likely due to the outdoor lifestyle and ectoparasite infestation of cats examined [18]. Similarly, *B. henselae* was more frequently retrieved than *B. clarridgeiae* in cat populations from other European countries (i.e. Cyprus, Portugal, Germany, Greece and Spain) [5, 22–24, 37]. Furthermore, the significantly higher prevalence of bacteraemia for *Bartonella* spp. in young cats (8.4%) compared to adults (1.8%) detected in this study is in line with previous findings [36, 38]. The significant difference in the prevalence of *Bartonella* spp. infection found across Italy (i.e. North, Central *vs* South regions) may be related to the different climate conditions among regions, which could have influenced the cat infection for this parasite [13, 36, 38, 39].

The high prevalence of haemoplasmas (11.6%) observed in this study is in line with results reported previously in owned cats from southern Italy (18.3%) [11], even if lower than that found in a stray cat population of northwestern Italy (31.3%) [40]. The significantly higher prevalence of infection by haemoplasmas in male cats is similar to that reported in previous studies, further indicating that sex and age may be risk factors for haemoplasma infections in cats [26, 41, 42]. The higher infection rate reported in older cats in these studies was not found to be significant in the data presented here, although cats older than 6 years displayed a higher infection rate (12%) than younger cats (8.4%), presumably because of the increasing risk of acquiring chronic subclinical infection over their lifetime [27]. Furthermore, no significant differences were found in the regional prevalence of haemoplasma infection in cats of the Italian Peninsula. Overall, the prevalence of haemoplasma infection found in this study was lower than that recorded in stray cats from different regions of Italy, where up to 16.7% and 1.3% were recorded for “*Ca.* Mycoplasma haemominutum”, and for *M. haemofelis* and “*Ca.* Mycoplasma turicensis” [18, 26, 40, 41] respectively, suggesting that cats with an outdoor lifestyle are at higher risk of haemoplasma infection. Furthermore, the association between FIV and haemoplasma infection revealed in this study, was concordant with those reported in several studies for cats population from different European countries (Italy, Spain, Portugal, Serbia, Cyprus) [5, 41, 43–45] suggesting that animals infected with these retroviruses were more susceptible to haemoplasma infection than the FIV-negative cats.

The absence of *Hepatozoon* and *Babesia* in cats tested might be a consequence of a non-exposure to infected ticks. This result was expected, even if *Babesia microti* has been previously detected by serology (20.3%) and molecularly (0.8%) in southern [11, 18] and northern Italy [46], respectively. No infection with other *Babesia* spp. was detected before in cats living in central and southern Italy [11, 19]. Differently, a high infection level (up to 8.1%) was observed for other *Babesia* spp. (i.e. *Babesia vogeli* and *Babesia canis*) in cat populations from Portugal [24, 47]. Similarly, the fact that no *Hepatozoon* DNA was amplified in this study was not surprising as this infection is not common within outdoor feline population of Italy [11]. *Hepatozoon* spp. are sometimes found in areas with high tick exposure, e.g. a low molecular prevalence (0.3–4.1%) detected for *Hepatozoon felis* in outdoor cats from Sicily [15] and few cases of *H. felis*, *H. canis* and *Hepatozoon silvestris* infections in cats in southern Italy [48]. In contrast, the overall molecular prevalence of *H. felis* infection was found to be much higher in other countries such as Spain, Portugal, Cyprus (from 1.6% to 37.9%) [5, 22, 24, 47, 49]. The lack of *Ehrlichia*/*Anaplasma* spp. in cats tested was concordant with previous studies, where no DNA was amplified from animals living in southern Italy [11, 15, 18], and with only one cat positive for *A. phagocytophilum* in the north of the country [46]. Conversely, antibody prevalence of up to 26.9% for *A. phagocytophilum* and 16.2% for *E. canis* was recorded in stray and/or outdoor cats from central and southern Italy [11, 18, 21]. A similar discrepancy in the molecular *versus* serological detection of *Ehrlichia* and *Anaplasma* spp. was described in feline populations from other countries of Europe (i.e. Spain, Portugal, Germany and Greece) [22, 24, 37, 47, 50]. Indeed, whilst DNA detection seems to be infrequent, with only *A. phagocytophilum* and *A. platys* DNA occasionally amplified, an antibody prevalence of *A. phagocytophilum* ranging from 2 to 8% has been reported in cats in Spain [50, 51], 13.5% in southern Portugal [52], 16.2% in Germany [53] and 22.1% in Sweden [54]. Even though no filarial DNA was amplified in the present study, this does not exclude the risk of *Dirofilaria* spp. infection, as they are known to occur in dogs and cats in different Italian regions [55].

## Conclusions

Although this study presents some limitations due to the lack of information on the health status and ectoparasitic treatments in the enrolled cats, data presented indicate that FeVBPs and haemoplasmas should be more investigated in privately-owned cats. Particular attention should be paid to *Bartonella* spp. infections, especially by *B. henselae*, which causes cat-scratch disease in humans [13, 56]. Furthermore, diseases associated with latent haemoplasma infections in both healthy and immunocompromised human patients are of emerging concern. In particular, *M. haemofelis* was detected in an immunodeficiency virus-infected human from Brazil who was co-infected with *B. henselae*, suggesting that *M. haemofelis* may have zoonotic potential [57]. Consequently, preventive measures against ectoparasites, for non-infected cats as well as infected cats after an appropriate treatment need to be implemented to protect both animals and humans living in the same environment. Increased awareness regarding both FeVBPs and haemoplasmas in cats is advocated.

## Data Availability

All data supporting the conclusions of this article are included within the article.

## Abbreviations

FeVBPs: feline vector-borne pathogens
VBP: vector-borne pathogen
VBDs: vector-borne diseases
FeLV: feline leukemia virus
FIV: feline immunodeficiency virus
cPCR: conventional PCR
*cox*1: cytochrome *c* oxidase subunit 1
CI: confidence interval
